# Altered microRNA expression in severe COVID‐19: Potential prognostic and pathophysiological role

**DOI:** 10.1002/ctm2.899

**Published:** 2022-06-13

**Authors:** Nathalie Garnier, Kato Pollet, Marie Fourcot, Morgan Caplan, Guillemette Marot, Julien Goutay, Julien Labreuche, Fabrice Soncin, Rabah Boukherroub, Didier Hober, Lille COVID Research Network (LICORNE), Sabine Szunerits, Julien Poissy, Ilka Engelmann

**Affiliations:** ^1^ Univ Lille, CHU Lille, Laboratoire de Virologie ULR3610 Lille France; ^2^ Univ Lille, CNRS, Centrale Lille Univ. Polytechnique Hauts‐de‐France, UMR 8520 ‐ IEMN Lille France; ^3^ Univ. Lille, CNRS, Inserm, CHU Lille Institut Pasteur de Lille, US 41 ‐ UAR 2014 ‐ PLBS Lille France; ^4^ CHU de Lille Pôle de réanimation Lille France; ^5^ Univ Lille, CHU Lille, ULR 2694 – METRICS Lille France; ^6^ Department of Biostatistics CHU Lille Lille France; ^7^ CNRS/IIS/Centre Oscar Lambret/Lille University SMMiL‐E Project, CNRS Délégation Hauts‐de‐France Lille France; ^8^ Laboratory for Integrated Micro Mechatronic Systems, Institute of Industrial Science, University of Tokyo,CNRS IRL2820 Tokyo Japan; ^9^ CNRS, UMR 8576‐UGSF‐Unité de Glycobiologie Structurale et Fonctionnelle, Inserm U1285, Univ Lille, CHU Lille Lille France

Dear Editor,

The pathophysiology of severe acute respiratory syndrome coronavirus 2 (SARS‐CoV‐2) infection is beginning to be elucidated but the role of microRNAs (miRNAs), small non‐coding RNAs that regulate gene expression, remains incompletely understood. In order to shed light on the role of miRNAs in the pathophysiology of SARS‐CoV‐2 infection, we have analyzed microRNA (miRNA) expression in nasopharyngeal swabs of coronavirus disease 2019 (COVID‐19) patients. We found that miRNA expression was globally reduced in severe COVID‐19 and identified several miRNAs of interest to discriminate severe and non‐severe COVID‐19. Our results suggest that these miRNAs are involved in the pathophysiology of COVID‐19 and represent not only promising biomarkers but also possible targets for antiviral or anti‐inflammatory treatment strategies.

miRNA expression was measured in nasopharyngeal swab specimens from patients with severe, non‐severe COVID‐19 and controls (Table S1). The number of expressed miRNAs was lower in the severe (median: 151) compared to the other groups (medians non‐severe: 184; controls: 190.5) (Figure 1A). Univariate analyses revealed that 14 miRNAs were differentially expressed in severe COVID‐19 versus controls (Table 1) and five of these, namely hsa‐miR‐125a‐5p, hsa‐miR‐200b‐3p, hsa‐miR‐340‐5p, hsa‐miR‐455‐5p and hsa‐miR‐491‐5p, were also down‐regulated in the severe versus the non‐severe COVID‐19 groups (Figure 1B, panels a,b; Table 1). A paralleled down‐regulation of miRNA expression in severe COVID‐19 was reported lately.^1^ RNA silencing pathways have been identified as antiviral defense mechanisms in plants and insects, and possibly in mammalian cells.^2^ To escape this antiviral defense, plant and insect viruses possess virus‐encoded suppressors of RNA interference, and this may extend to mammalian cells.^3^ An interesting hypothesis to explain our findings is thus that SARS‐CoV‐2 targets cellular miRNA biogenesis. In line with this, expression of the RNA interference machinery components Ago2, Dicer and Drosha was significantly down‐regulated in COVID‐19 patients.^4^ Furthermore, miRNA depletion enhances proinflammatory cytokine production, including expression of interleukin‐6 (IL‐6).^5^ The global repression of miRNA expression in severe COVID‐19 that we observed here may thus be causally linked to the hyperinflammatory state found in severe COVID‐19.

**FIGURE 1:**
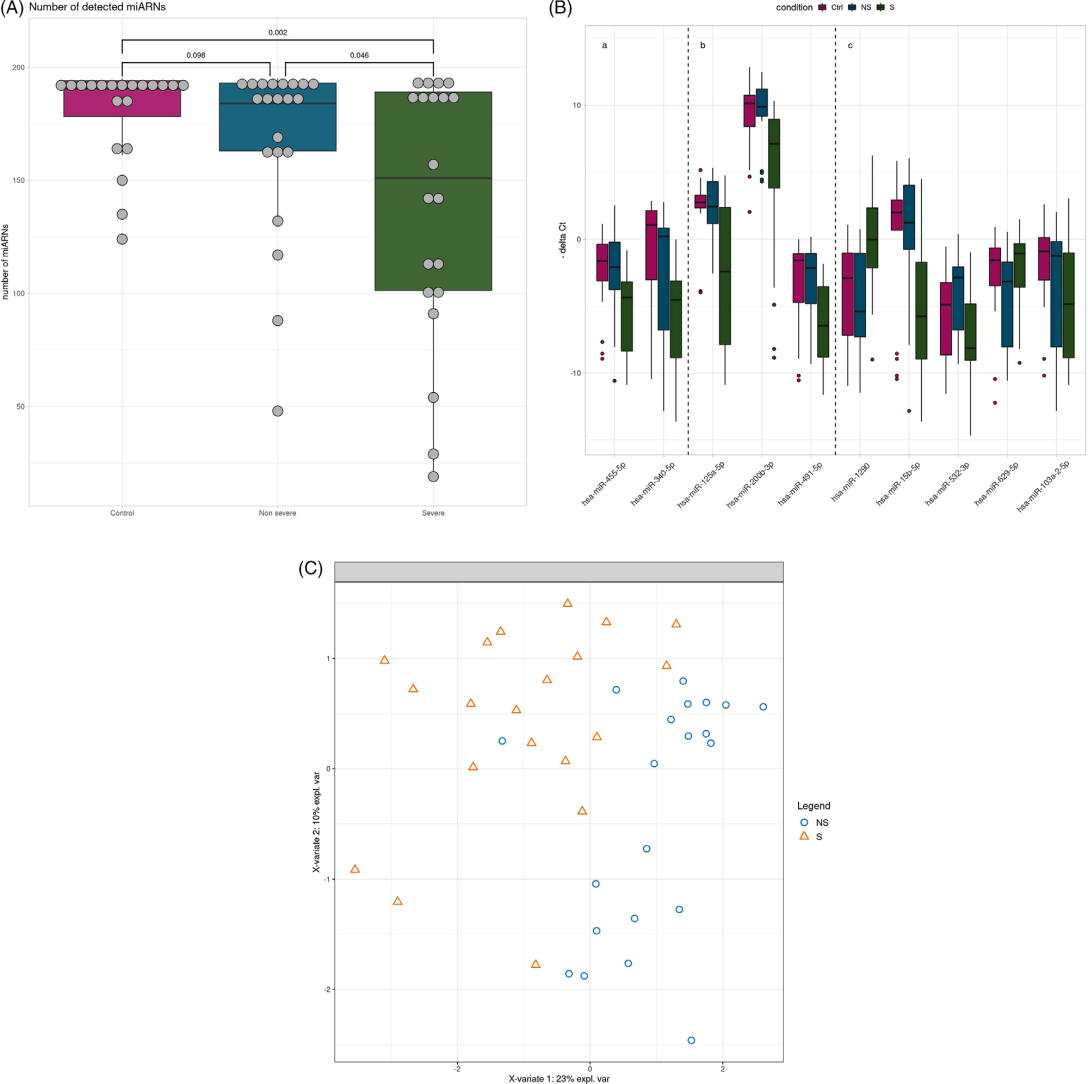
miRNA expression in non‐severe compared to severe COVID‐19. (A) Number of miRNAs detected in nasopharyngeal swab specimens of severe (*n* = 20), non‐severe COVID‐19 (*n* = 21) and controls (*n* = 20). Box plots showing median and interquartile ranges of the number of expressed miRNAs in each group. (B) Box‐plots showing the expression of miRNAs that are differentially expressed between severe (S) and non‐severe (NS) COVID‐19 and control groups (Ctrl) in univariate analyses (panels Ba,b). Box‐plots showing the expression of miRNAs that discriminate severe and non‐severe COVID‐19 by the sPLS‐DA multivariate analysis (panels Bb,c). The miRNAs identified by both analyses are shown in panel Bb. The bars show the medians and the boxes the interquartile ranges of normalized miRNA expression. (C) Severe (S, triangles) and non‐severe COVID‐19 cases (NS, circles) are discriminated by miRNA expression by sPLS‐DA analysis

**TABLE 1:** miRNAs differentially expressed between severe COVID‐19 and controls and non‐severe COVID‐19

miRNA	Log_2_ fold‐change severe COVID‐19 versus control	Adjusted *p*‐value	Log_2_ fold‐change severe COVID‐19 versus non‐severe COVID‐19	Adjusted *p*‐value
hsa‐miR‐125a‐5p	−5.170	.044	−4.853	.045
hsa‐miR‐200b‐3p	−3.012	.044	−2.752	.013
hsa‐miR‐200c‐3p	−2.328	.044	−1.280	NS
hsa‐miR‐218‐5p	−6.534	.044	−5.165	NS
hsa‐miR‐27a‐3p	−2.848	.044	−1.790	NS
hsa‐miR‐30c‐5p	−8.041	.044	−7.232	NS
hsa‐miR‐30d‐5p	−3.327	.044	−1.717	NS
hsa‐miR‐375	−1.977	.044	−0.859	NS
hsa‐miR‐378a‐3p	−4.365	<.001	−1.919	NS
hsa‐miR‐422a	−5.682	.002	0.067	NS
hsa‐miR‐455‐5p	−2.724	.044	−2.260	.034
hsa‐miR‐532‐5p	−1.898	.044	−0.499	NS
hsa‐miR‐340‐5p	−5.601	.044	−4.744	.011
hsa‐miR‐491‐5p	−4.887	.044	−4.326	.031

Abbreviation: NS, not significant.

Multivariate sparse Partial Least Squares‐Discriminant Analysis (sPLS‐DA) analysis selected eight miRNAs, hsa‐miR‐125a‐5p, hsa‐miR‐1290, hsa‐miR‐15b‐5p, hsa‐miR‐491‐5p, hsa‐miR‐532‐3p, hsa‐miR‐200b‐3p, hsa‐miR‐629‐5p and hsa‐miR‐103a‐2‐5p, that discriminated severe and the non‐severe COVID‐19 (Figure 1B, panels b,c). Five of these miRNAs were not differentially expressed in univariate analyses (Figure 1B, panel c) but were necessary to build scores with linear discriminant analysis. When using leave‐one‐out cross validation, 35 out of 41 patients (85.37%) were correctly predicted (Table S2), suggesting that these miRNAs could be used in scores to cluster patients. The coefficients of these scores should be built from a future study that includes more patients and validated in an independent cohort.

The comparison of results of differential expression analysis and sPLS‐DA revealed three miRNA in common, namely hsa‐miR‐125a‐5p, hsa‐miR‐491‐5p and hsa‐miR‐200b‐3p (Figure 1B, panel b). These miRNAs discriminated severe from non‐severe cases with areas under the curve ranging from 0.76 to 0.79 (Figure 2). To evaluate their performance as biomarkers to predict disease severity, it would be interesting to determine their expression at different time points, that is, before, during and after resolution of severe COVID‐19.

**FIGURE 2:**
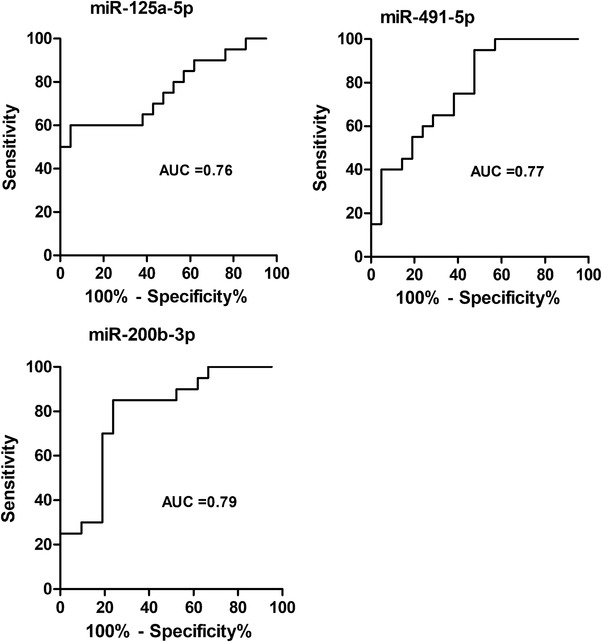
Receiver operator characteristic (ROC) analysis for the discrimination of severe and non‐severe COVID‐19. ROC curve and area under the ROC curve (AUC) values for the classification into severe and non‐severe COVID‐19 based on miRNA expression are shown for the three miRNAs identified in common by sPLS‐DA and univariate analysis

We hypothesized that the 10 miRNAs selected by sPLS‐DA and/or differential analysis (Figure 1B) play a role in the pathophysiology of severe COVID‐19. Ninety‐five validated target genes were retrieved (Table S3). Gene ontology (GO) enrichment analysis revealed enrichment for five, eight and zero GO terms associated with biological process, molecular function and cellular component, respectively (Tables S4 and S5). Enriched biological processes were involved in deoxyribonucleid acid (DNA) damage, ubiquitination and antigen processing and presentation (Table S4). Enriched molecular functions were involved in protein kinase activity, ubiquitination and RNA polymerase II activity (Table S5). Kegg pathway analysis revealed enrichment in pathways that play a role in ubiquitination, viral infections and the immune response (Table 2). Reactome pathway analysis showed enrichment for pathways involved in antigen processing, NFkappaB and other signaling pathways (Table 2).

**TABLE 2:** Kegg and reactome pathways enrichment analysis

Kegg pathway	*p*‐Value	Adjusted *p*‐value (BH^*^)
hsa04120_ubiquitin_mediated_proteolysis	.001	.004
hsa05162_measles	.001	.004
hsa05169_Epstein‐Barr_virus_infection	.001	.004
hsa05170_human_immunodeficiency_virus_1_infection	.002	.004
hsa04218_cellular_senescence	.002	.004
hsa04630_JAK‐STAT_signaling_pathway	.003	.004
hsa05166_human_T‐cell_leukemia_virus_1_infection	.008	.012
hsa05131_shigellosis	.011	.014
hsa05200_pathways_in_cancer	.032	.035

Reactome pathway	*p*‐Value	Adjusted *p*‐value (BH^*^)
R‐HSA‐983168_antigen processing: ubiquitination and proteasome degradation	<.001	<.001
R‐HSA‐2871837_FCERI mediated NF‐kB activation	<.001	<.001
R‐HSA‐5607764_CLEC7A (Dectin‐1) signaling	<.001	<.001
R‐HSA‐9020702_interleukin‐1 signaling	<.001	<.001
R‐HSA‐202424_downstream TCR signaling	<.001	<.001

* BH: Benjamini‐Hochberg false discovery rate correction

Of the miRNAs found of interest to discriminate between severe and non‐severe COVID‐19 in our study, some were reported to be implicated in viral and other infections: miR‐455‐5p was up‐regulated in rabies virus infection in vitro, decreased suppressor of cytokine signaling 3 (SOCS3) expression and increased signal transducer and activator of transcription 3 (STAT3) activity, resulting in enhanced viral replication and the production of IL‐6.^6^ Furthermore, this miRNA targeted the C‐C motif chemokine receptor 5 (CCR5).^7^ Of interest, CCR5 is involved in severe COVID‐19 and has been proposed as anti‐inflammatory treatment target.^8^ Taken together, this underlines the role of hsa‐miR‐455‐5p in viral infections and the inflammatory response and its potential as target of therapeutic interventions.

hsa‐miR‐532‐3p diminished the levels of ASK1 and downstream phosphorylation/translocation of p38 MAPK during lipopolysaccharide (LPS)/ tumor necrosis factor‐α (TNF‐α)‐induced inflammation in macrophages and reduced the release of various pro‐inflammatory cytokines and chemokines, including IL‐6 and TNF‐alpha.^9^ IL‐6 is a proinflammatory cytokine that has been reported to be involved in the cytokine storm observed in severe COVID‐19.^10^ The down‐regulation of anti‐inflammatory miRNAs, such as hsa‐miR‐532‐3p, hsa‐miR‐340‐5p and hsa‐miR‐455‐5p, in severe COVID‐19 (Table 1) is in line with a hyperinflammatory state in severe COVID‐19. Supplementation of these anti‐inflammatory miRNAs may represent a novel therapeutic strategy.

Recently, direct and indirect miRNA interactions with other miRNAs have been described (reviewed^11^). Therefore, it cannot be excluded that the miRNAs identified in this study directly or indirectly influence expression of other miRNAs and thus have a broad impact on miRNA and gene expression.

We next searched for miRNAs that may directly target the SARS‐CoV‐2 genome among the miRNAs associated with severe COVID‐19. We found that hsa‐miR‐15b‐5p was predicted to bind to the SARS‐CoV‐2 genome at 16 positions (Table S6). Furthermore, hsa‐miR‐15b‐5p was confirmed to interact with SARS‐CoV‐2 RNA in vitro.^12^ hsa‐miR‐15b‐5p was down‐regulated in severe COVID‐19 in our study (Figure 1B, panel c) and in the lungs of hamsters infected with SARS‐CoV‐2.^13^ Taken together, this suggests that the down‐regulation of hsa‐miR‐15b‐5p may represent a mechanism of SARS‐CoV‐2 to escape the host antiviral defense.

In conclusion, our analysis of miRNA expression in nasopharyngeal swabs revealed a general reduction of miRNA expression in severe COVID‐19 patients. Several miRNAs of interest to discriminate severe and non‐severe COVID‐19 were identified. Functional analysis of these miRNAs suggested a role in the pathophysiology of the disease. Further characterization of their implication in SARS‐CoV‐2 infection will enable elucidation of the molecular mechanisms and may reveal potential targets for antiviral or anti‐inflammatory treatment of COVID‐19.

## CONFLICT OF INTEREST

The authors declare no conflict of interest.

## Supporting information

Table S1. Patients’ characteristics
Table S2. Confusion matrix for prediction of COVID‐19 severity from miRNA expression with linear discriminant analysis
Table S3. Target genes
Table S4. GO biological processes
Table S5. GO molecular functions
Table S6. miRDB prediction of miRNAs binding to SARS‐CoV‐2 genome
