# Supplementary material for: Altered microRNA expression in severe COVID‐19: Potential prognostic and pathophysiological role
Source: Clin Transl Med. 2022 Jun 13;12(6):e899. doi: 10.1002/ctm2.899 (PMC9191908; doi:10.1002/ctm2.899)
Supplement: Supplementary file 1 — Table S1. Patients’ characteristics Table S2. Confusion matrix for prediction of COVID‐19 severity from miRNA expression with linear discriminant analysis Table S3. Target genes Table S4. GO biological processes Table S5. GO molecular functions Table S6. miRDB prediction of miRNAs binding to SARS‐CoV‐2 genome [file CTM2-12-0-s001.docx]

**Supplementary Methods**

**Patients and specimens**

A total of 61 patients were included in the study, including 20 patients with severe COVID-19, 21 patients with non-severe COVID-19 and 20 controls (Supplementary table 1). Severe COVID-19 patients were patients admitted to the intensive care unit for oxygen treatment. Non-severe COVID-19 patients were seen in ambulatory medicine and not hospitalized, without requirement of oxygen treatment. All COVID-19 patients had SARS-CoV-2 detected by RT-PCR. The control group consisted of 20 patients that had nasopharyngeal swab specimens performed for routine diagnostic purposes and who did not have COVID-19. In control patients that were recruited during the same time period as the COVID-19 patients, COVID-19 was ruled out by clinical criteria and negative SARS-CoV-2 RT-PCR results in at least two specimens (n=15). Additional controls that were from March 2019, i.e. before the emergence of SARS-CoV-2, had no SARS-CoV-2 RT-PCR performed (n=5). Nasopharyngeal swab specimens were obtained from all patients for routine diagnostic purposes by using flocked swabs that were placed in universal transport medium. Specimens were stored at -80°C. The first available specimen was used and median time of sampling was 7 days after symptom onset (Supplementary Table 1). Demographic data were retrospectively collected from hospital charts and the virology laboratory database. The study was approved by the French Institutional Authority for Personal Data Protection (Commission Nationale de l’Informatique et des Libertés DR-2020-178, October 22nd, 2020) and the ethics committee (Comité de Protection des Personnes Nord Ouest IV, ECH20/09, September 7th, 2020).

**RNA extraction**

RNA extraction was performed by using the MagMAX mirVana Total RNA Isolation Kit (Thermofisher Scientific, Courtaboeuf, France) according to the manufacturer’s instructions.

**miRNA profiling**

Poly-A-tailing, adapter ligation, reverse transcription and pre-amplification was performed using the [TaqMan Advanced miRNA cDNA Synthesis Kit](https://www.thermofisher.com/order/catalog/product/A28007)  (Thermofisher Scientific, Courtaboeuf, France) according to the manufacturer’s instructions. Quantitative PCR was done with TaqMan Fast Advanced Master Mix and the TaqMan™ Advanced miRNA Human A and B Cards (Thermofisher Scientific, Courtaboeuf, France) on a QuantStudio 7 Flex instrument (Thermofisher Scientific, Courtaboeuf, France) according to the manufacturer’s instructions, allowing to quantify the expression of 384 miRNAs per card. The expression of miRNAs was quantified using the online application of the Thermofisher Cloud ([www.thermofisher.com](http://www.thermofisher.com/)). Amplification data were reviewed and the thresholds adapted.

In the following of this paper a miRNA is called “detected” if its Ct-value was strictly lower than 40. The threshold was chosen because the PCR contains 40 amplification cycles. A specimen with no amplification is set to Ct=40 by the Thermofisher Cloud application for relative quantification. Therefore, any miRNA giving a signal at a Ct lower than 40 is “detected”. We considered as “expressed” a miRNA detected in at least 80% of the nasopharyngeal swab specimens of at least one of the three groups. Only the expressed miRNAs were kept for further analyses

**Target prediction, GO enrichment and pathway analysis**

miRWalk (http://mirwalk.umm.uni-heidelberg.de/) was used for the prediction of targets of dysregulated miRNAs and GO enrichment analysis, Kegg and Reactome pathways enrichment analyses [1]. Only validated target genes were considered for GO enrichment analysis, Kegg and Reactome pathways. An adjusted p-value <0.05 was considered statistically significant. miRDB was used for the prediction of miRNAs that target the SARS-CoV-2 genome (NC_045512.2) [2]. Only miRNAs with a target score of at least 90 were considered.

**Statistical analysis**

Statistical analysis was performed by using R (version 4.1.2). Following the idea of global mean normalization [3] but reproducing the Thermofisher cloud normalization, the data were normalized by subtracting from each Ct value the median Ct value of the plate from which it originated. The delta delta Ct method was used to calculate fold changes of miRNA expression between groups [4].

To compare the number of expressed miRNAs between groups, a Kruskal-Wallis test was performed followed by Dunn’s post-hoc test. Then differential expression analysis of miRNA between the severe COVID-19 group and the control group were performed using Mann-Whitney U tests. Raw p-values of these multiple univariate analyses were adjusted with Benjamini-Hochberg method [5], which controls the False Discovery Rate. miRNAs presenting an adjusted p-value of <0.05 were considered differentially expressed between the severe COVID-19 group and the control group. Expression of these differentially expressed miRNAs was then compared between severe and non-severe COVID-19 groups by using Mann-Whitney U tests and Bonferroni correction to control the Family-Wise Error Rate (Probability of having at least one false positive). An adjusted p-value of <0.05 was considered statistically significant.

In parallel to these univariate analyses sparse Partial Least Squares-Discriminant Analysis (sPLS-DA) was performed with the mixOmics package [6]. This multivariate analysis enables to discriminate severe and non-severe COVID-19 groups. It is appropriate to this dataset which contains much more variables than individuals as PLS can deal with correlated variables and sparsity ensures variable selection. The number of components was set to 2 in order to facilitate visualization.

Linear discriminant analyses were run with the lda function of the MASS R package on the subset of miRNAs selected by sPLS-DA. Leave-one-out cross validation was used to predict the severity of each COVID-19 patient from these 8 miRNAs. A confusion matrix was built to compare the prediction with the true group and evaluate the potential of prediction of these candidate biomarkers.

Receiver operating characteristic (ROC) analysis with area under the curve (AUC) was performed to analyze the diagnostic performance to differentiate severe and non-severe COVID-19 of promising miRNAs defined by the consensus between differential expression analysis and sPLS-DA.

**Supplementary Table 1. Patients’ characteristics**

|  | **COVID-19** | | **Control** |
| --- | --- | --- | --- |
|  | Non severe | Severe |  |
| Number of cases | 21 | 20 | 20 |
| M/F | 10/11 | 14/6 | 9/11 |
| Age (median (range)) | 30 (23 – 62) | 60 (29 – 78) | 71 (23 – 89) |
| Days post symptom onset (median) | 7 | 7 | NA |
| Hospital admission, n (%) | 0 | 20 (100%) | 19 (95%) |
|  |  |  |  |
| **Biologic tests;** median (n with available data) |  |  |  |
| Hemoglobin (g/dL) | NA | 13.5 (n=20) | 12.4 (n=18) |
| Platelets (10^9^/L) | NA | 197 (n=20) | 220 (n=18) |
| Leukocytes (10^9^/L) | NA | 6.2 (n=20) | 9.8 (n=18) |
| CRP (mg/L) | NA | 93 (n=20) | 89 (n=19) |
| LDH (U/L) | NA | 449 (n=15) | 262 (n=5) |
|  |  |  |  |
| **ICU characteristics** |  |  |  |
|  |  |  |  |
| Intensive care unit stay, n (%) | 0 | 20 (100%) | 5 (25%) |
| Oxygen treatment, n (%) | 0 | 20 (100%) | 7 (35%) |
| SOFA^†^ score at admission (median) | NA | 6.5 | 2 |
| Score WHO^‡^ at admission (median) | NA | 6 | NA |
| Score WHO^‡^ day 28 (median) | NA | 4 (n=16) | NA |
| Oxygen by mask high concentration, n (%) | NA | 9 (45%) | 1 (2.5%) |
| Optiflow, n (%) | NA | 13 (65%) | 1 (2.5%) |
| Non-invasive ventilation, n (%) | NA | 9 (45%) | 2 (5%) |
| Invasive mechanical ventilation, n (%) | NA | 16 (80%) | 2 (5%) |
| Extracorporeal membrane oxygenation, n (%) | NA | 1 (5%) | 0 |
|  |  |  |  |
| **Underlying diseases**, n (%) |  |  |  |
| Kidney disease | 0 | 1 (5%) | 3 (15%) |
| Hypertension | 3 (14%) | 9 (45%) | 9 (45%) |
| Cardiovascular diseases | 2 (10%) | 3 (15%) | 8 (40%) |
| Respiratory system disease | 3 (14%) | 5 (25%) | 6 (30%) |
| Diabetes | 0 | 3 (15%) | 6 (30%) |
| Cancer | 0 | 0 | 2 (10%) |
| Malignant haemopathy | 0 | 1 (5%) | 1 (5%) |
| Immunosuppression | 1 (5%) | 1 (5%) | 3 (15%) |
|  |  |  |  |

^†^SOFA: Sequential Organ Failure Assessment Score

^‡^WHO: world health organization

**Supplementary Table 2. Confusion matrix for prediction of COVID-19 severity from miRNA expression with linear discriminant analysis**

|  | **Predicted as non-severe COVID-19** | **Predicted as severe COVID-19** |
| --- | --- | --- |
| **non-severe COVID-19 (n=21)** | 19 | 2 |
| **severe COVID-19 (n=20)** | 4 | 16 |

**Supplementary Table 3. Target genes**

| Entrezid | Genesymbol | Description |
| --- | --- | --- |
| 6310 | ATXN1 | Homo sapiens ataxin 1 (ATXN1), transcript variant 1, mRNA. |
| 596 | BCL2 | Homo sapiens BCL2 apoptosis regulator (BCL2), transcript variantalpha, mRNA. |
| 490 | ATP2B1 | Homo sapiens ATPase plasma membrane Ca2+ transporting 1 (ATP2B1),transcript variant 1, mRNA. |
| 59338 | PLEKHA1 | Homo sapiens pleckstrin homology domain containing A1 (PLEKHA1),transcript variant 2, mRNA. |
| 23131 | GPATCH8 | Homo sapiens G-patch domain containing 8 (GPATCH8), transcriptvariant 1, mRNA. |
| 51114 | ZDHHC9 | Homo sapiens zinc finger DHHC-type palmitoyltransferase 9 (ZDHHC9),transcript variant 2, mRNA. |
| 605 | BCL7A | Homo sapiens BAF chromatin remodeling complex subunit BCL7A(BCL7A), transcript variant 2, mRNA. |
| 163 | AP2B1 | Homo sapiens adaptor related protein complex 2 subunit beta 1(AP2B1), transcript variant 1, mRNA. |
| 91746 | YTHDC1 | Homo sapiens YTH domain containing 1 (YTHDC1), transcript variant1, mRNA. |
| 6812 | STXBP1 | Homo sapiens syntaxin binding protein 1 (STXBP1), transcriptvariant 2, mRNA. |
| 7335 | UBE2V1 | Homo sapiens ubiquitin conjugating enzyme E2 V1 (UBE2V1),transcript variant 4, mRNA. |
| 221937 | FOXK1 | Homo sapiens forkhead box K1 (FOXK1), mRNA. |
| 56990 | CDC42SE2 | Homo sapiens CDC42 small effector 2 (CDC42SE2), transcript variant2, mRNA. |
| 3955 | LFNG | Homo sapiens LFNG O-fucosylpeptide3-beta-N-acetylglucosaminyltransferase (LFNG), transcript variant |
| 440193 | CCDC88C | Homo sapiens coiled-coil domain containing 88C (CCDC88C), mRNA. |
| 25994 | HIGD1A | Homo sapiens HIG1 hypoxia inducible domain family member 1A(HIGD1A), transcript variant 1, mRNA; nuclear gene for |
| 90809 | PIP4P1 | Homo sapiens phosphatidylinositol-4,5-bisphosphate 4-phosphatase 1(PIP4P1), transcript variant 1, mRNA. |
| 7763 | ZFAND5 | Homo sapiens zinc finger AN1-type containing 5 (ZFAND5), transcriptvariant a, mRNA. |
| 996 | CDC27 | Homo sapiens cell division cycle 27 (CDC27), transcript variant 1,mRNA. |
| 1111 | CHEK1 | Homo sapiens checkpoint kinase 1 (CHEK1), transcript variant 2,mRNA |
| 5255 | PHKA1 | Homo sapiens phosphorylase kinase regulatory subunit alpha 1(PHKA1), transcript variant 2, mRNA. |
| 3977 | LIFR | Homo sapiens LIF receptor subunit alpha (LIFR), transcript variant1, mRNA. |
| 813 | CALU | Homo sapiens calumenin (CALU), transcript variant 2, mRNA. |
| 9209 | LRRFIP2 | Homo sapiens LRR binding FLII interacting protein 2 (LRRFIP2),transcript variant 3, mRNA. |
| 64225 | ATL2 | Homo sapiens atlastin GTPase 2 (ATL2), transcript variant 2, mRNA. |
| 27 | ABL2 | Homo sapiens ABL proto-oncogene 2, non-receptor tyrosine kinase(ABL2), transcript variant d, mRNA. |
| 896 | CCND3 | Homo sapiens cyclin D3 (CCND3), transcript variant 1, mRNA. |
| 552889 | ATXN7L3B | Homo sapiens ataxin 7 like 3B (ATXN7L3B), mRNA. |
| 2113 | ETS1 | Homo sapiens ETS proto-oncogene 1, transcription factor (ETS1),transcript variant 1, mRNA. |
| 7465 | WEE1 | Homo sapiens WEE1 G2 checkpoint kinase (WEE1), transcript variant2, mRNA. |
| 8867 | SYNJ1 | Homo sapiens synaptojanin 1 (SYNJ1), transcript variant 3, mRNA. |
| 651746 | ANKRD33B | Homo sapiens ankyrin repeat domain 33B (ANKRD33B), mRNA. |
| 11228 | RASSF8 | Homo sapiens Ras association domain family member 8 (RASSF8),transcript variant 1, mRNA. |
| 55206 | SBNO1 | Homo sapiens strawberry notch homolog 1 (SBNO1), transcript variant1, mRNA. |
| 63967 | CLSPN | Homo sapiens claspin (CLSPN), transcript variant 2, mRNA. |
| 4781 | NFIB | Homo sapiens nuclear factor I B (NFIB), transcript variant 1, mRNA. |
| 79751 | SLC25A22 | Homo sapiens solute carrier family 25 member 22 (SLC25A22),transcript variant 1, mRNA; nuclear gene for mitochondrial product. |
| 51444 | RNF138 | Homo sapiens ring finger protein 138 (RNF138), transcript variant3, mRNA. |
| 7049 | TGFBR3 | Homo sapiens transforming growth factor beta receptor 3 (TGFBR3),transcript variant 2, mRNA. |
| 862 | RUNX1T1 | Homo sapiens RUNX1 partner transcriptional co-repressor 1(RUNX1T1), transcript variant 5, mRNA. |
| 5451 | POU2F1 | Homo sapiens POU class 2 homeobox 1 (POU2F1), transcript variant 2,mRNA. |
| 83607 | AMMECR1L | Homo sapiens AMMECR1 like (AMMECR1L), transcript variant 2, mRNA. |
| 57154 | SMURF1 | Homo sapiens SMAD specific E3 ubiquitin protein ligase 1 (SMURF1),transcript variant 3, mRNA. |
| 9354 | UBE4A | Homo sapiens ubiquitination factor E4A (UBE4A), transcript variant2, mRNA. |
| 10000 | AKT3 | Homo sapiens AKT serine/threonine kinase 3 (AKT3), transcriptvariant 3, mRNA. |
| 905 | CCNT2 | Homo sapiens cyclin T2 (CCNT2), transcript variant a, mRNA. |
| 5110 | PCMT1 | Homo sapiens protein-L-isoaspartate (D-aspartate)O-methyltransferase (PCMT1), transcript variant 2, mRNA. |
| 55334 | SLC39A9 | Homo sapiens solute carrier family 39 member 9 (SLC39A9),transcript variant 2, mRNA. |
| 10802 | SEC24A | Homo sapiens SEC24 homolog A, COPII coat complex component(SEC24A), transcript variant 2, mRNA. |
| 390 | RND3 | Homo sapiens Rho family GTPase 3 (RND3), transcript variant 1,mRNA. |
| 8945 | BTRC | Homo sapiens beta-transducin repeat containing E3 ubiquitin proteinligase (BTRC), transcript variant 3, mRNA. |
| 92 | ACVR2A | Homo sapiens activin A receptor type 2A (ACVR2A), transcriptvariant 1, mRNA. |
| 10558 | SPTLC1 | Homo sapiens serine palmitoyltransferase long chain base subunit 1(SPTLC1), transcript variant 3, mRNA. |
| 7227 | TRPS1 | Homo sapiens transcriptional repressor GATA binding 1 (TRPS1),transcript variant 2, mRNA. |
| 10152 | ABI2 | Homo sapiens abl interactor 2 (ABI2), transcript variant 1, mRNA. |
| 23271 | CAMSAP2 | Homo sapiens calmodulin regulated spectrin associated proteinfamily member 2 (CAMSAP2), transcript variant 1, mRNA. |
| 9444 | QKI | Homo sapiens QKI, KH domain containing RNA binding (QKI),transcript variant 5, mRNA. |
| 51621 | KLF13 | Homo sapiens Kruppel like factor 13 (KLF13), transcript variant 2,mRNA. |
| 22880 | MORC2 | Homo sapiens MORC family CW-type zinc finger 2 (MORC2), transcriptvariant 1, mRNA. |
| 23471 | TRAM1 | Homo sapiens translocation associated membrane protein 1 (TRAM1),transcript variant 2, mRNA. |
| 1788 | DNMT3A | Homo sapiens DNA methyltransferase 3 alpha (DNMT3A), transcriptvariant 5, mRNA. |
| 80851 | SH3BP5L | Homo sapiens SH3 binding domain protein 5 like (SH3BP5L),transcript variant 2, mRNA. |
| 57089 | ENTPD7 | Homo sapiens ectonucleoside triphosphate diphosphohydrolase 7(ENTPD7), transcript variant 1, mRNA. |
| 6774 | STAT3 | Homo sapiens signal transducer and activator of transcription 3(STAT3), transcript variant 4, mRNA. |
| 23030 | KDM4B | Homo sapiens lysine demethylase 4B (KDM4B), transcript variant 2,mRNA. |
| 64393 | ZMAT3 | Homo sapiens zinc finger matrin-type 3 (ZMAT3), transcript variant3, mRNA. |
| 2683 | B4GALT1 | Homo sapiens beta-1,4-galactosyltransferase 1 (B4GALT1), transcriptvariant 2, mRNA. |
| 1951 | CELSR3 | Homo sapiens cadherin EGF LAG seven-pass G-type receptor 3(CELSR3), mRNA. |
| 1810 | DR1 | Homo sapiens down-regulator of transcription 1 (DR1), mRNA. |
| 5713 | PSMD7 | Homo sapiens proteasome 26S subunit, non-ATPase 7 (PSMD7), mRNA. |
| 6664 | SOX11 | Homo sapiens SRY-box transcription factor 11 (SOX11), mRNA. |
| 8760 | CDS2 | Homo sapiens CDP-diacylglycerol synthase 2 (CDS2), mRNA. |
| 6197 | RPS6KA3 | Homo sapiens ribosomal protein S6 kinase A3 (RPS6KA3), mRNA. |
| 7189 | TRAF6 | Homo sapiens TNF receptor associated factor 6 (TRAF6), transcriptvariant 2, mRNA. |
| 9371 | KIF3B | Homo sapiens kinesin family member 3B (KIF3B), mRNA. |
| 1795 | DOCK3 | Homo sapiens dedicator of cytokinesis 3 (DOCK3), mRNA. |
| 1650 | DDOST | Homo sapiens dolichyl-diphosphooligosaccharide--proteinglycosyltransferase non-catalytic subunit (DDOST), mRNA. |
| 1820 | ARID3A | Homo sapiens AT-rich interaction domain 3A (ARID3A), mRNA. |
| 830 | CAPZA2 | Homo sapiens capping actin protein of muscle Z-line subunit alpha 2(CAPZA2), mRNA. |
| 10527 | IPO7 | Homo sapiens importin 7 (IPO7), mRNA. |
| 7832 | BTG2 | Homo sapiens BTG anti-proliferation factor 2 (BTG2), mRNA. |
| 5930 | RBBP6 | Homo sapiens RB binding protein 6, ubiquitin ligase (RBBP6),transcript variant 1, mRNA. |
| 6515 | SLC2A3 | Homo sapiens solute carrier family 2 member 3 (SLC2A3), mRNA. |
| 23011 | RAB21 | Homo sapiens RAB21, member RAS oncogene family (RAB21), mRNA. |
| 51592 | TRIM33 | Homo sapiens tripartite motif containing 33 (TRIM33), transcriptvariant a, mRNA. |
| 51132 | RLIM | Homo sapiens ring finger protein, LIM domain interacting (RLIM),transcript variant 1, mRNA. |
| 54887 | UHRF1BP1 | Homo sapiens UHRF1 binding protein 1 (UHRF1BP1), mRNA. |
| 55664 | CDC37L1 | Homo sapiens cell division cycle 37 like 1 (CDC37L1), mRNA. |
| 55824 | PAG1 | Homo sapiens phosphoprotein membrane anchor with glycosphingolipidmicrodomains 1 (PAG1), mRNA. |
| 56977 | STOX2 | Homo sapiens storkhead box 2 (STOX2), transcript variant 1, mRNA. |
| 57534 | MIB1 | Homo sapiens mindbomb E3 ubiquitin protein ligase 1 (MIB1), mRNA. |
| 57551 | TAOK1 | Homo sapiens TAO kinase 1 (TAOK1), transcript variant 1, mRNA. |
| 53354 | PANK1 | Homo sapiens pantothenate kinase 1 (PANK1), transcript variantgamma, mRNA. |
| 134353 | LSM11 | Homo sapiens LSM11, U7 small nuclear RNA associated (LSM11), mRNA. |
| 387522 | PEDS1-UBE2V1 | Homo sapiens PEDS1-UBE2V1 readthrough (PEDS1-UBE2V1), mRNA. |

**Supplementary Table 4. GO biological processes**

| Name | Pvalue | adjusted Pvalue(BH) |
| --- | --- | --- |
| GO:0000209_protein_polyubiquitination | <0.001 | <0.001 |
| GO:0006974_cellular_response_to_DNA_damage_stimulus | <0.001 | <0.001 |
| GO:0019886_antigen_processing_and_presentation_of_exogenous_peptide_antigen_via_MHC_class_II | <0.001 | 0.001 |
| GO:0006511_ubiquitin-dependent_protein_catabolic_process | 0.006 | 0.014 |
| GO:0035556_intracellular_signal_transduction | 0.019 | 0.037 |

**Supplementary Table 5. GO molecular functions**

| Name | Pvalue | adjusted Pvalue(BH) |
| --- | --- | --- |
| GO:0004672_protein_kinase_activity | <0.001 | 0.002 |
| GO:0030165_PDZ_domain_binding | <0.001 | 0.002 |
| GO:0004842_ubiquitin-protein_transferase_activity | <0.001 | 0.002 |
| GO:0061630_ubiquitin_protein_ligase_activity | 0.002 | 0.005 |
| GO:0019901_protein_kinase_binding | 0.006 | 0.011 |
| GO:0000978_RNA_polymerase_II_proximal_promoter_sequence-specific_DNA_binding | 0.026 | 0.040 |
| GO:0001228_DNA-binding_transcription_activator_activity_RNA_polymerase_II-specific | 0.043 | 0.048 |
| GO:0008134_transcription_factor_binding | 0.042 | 0.048 |

**Supplementary Table 6. miRDB prediction of miRNAs binding to SARS-CoV-2 genome**

| **miRNA Name** | **miRNA sequence** | **Target sequence** | **Target Score** | **Number of predicted binding positions** | **Predicted miRNA seed binding positions** |
| --- | --- | --- | --- | --- | --- |
| hsa-miR-15b-5p | UAGCAGCACAUCAUGGUUUACA | NC_045512.2 SARS-CoV-2 isolate Wuhan-Hu-1 | 99 | 16 | 3163; 5384; 8458; 8614; 13090; 14562; 14781; 19857; 24094; 24634; 25683; 26723; 28921; 28935; 28938; 29023 |

**Supplementary References**

1. Sticht C, Torre CDL, Parveen A, Gretz N (2018) miRWalk: An online resource for prediction of microRNA binding sites. PLOS ONE 13:e0206239. https://doi.org/10.1371/journal.pone.0206239

2. Chen Y, Wang X (2020) miRDB: an online database for prediction of functional microRNA targets. Nucleic Acids Res 48:D127–D131. https://doi.org/10.1093/nar/gkz757

3. Mestdagh P, Van Vlierberghe P, De Weer A, et al (2009) A novel and universal method for microRNA RT-qPCR data normalization. Genome Biology 10:R64. https://doi.org/10.1186/gb-2009-10-6-r64

4. Pfaffl MW (2001) A new mathematical model for relative quantification in real-time RT-PCR. Nucleic Acids Res 29:e45

5. Benjamini Y, Hochberg Y (1995) Controlling the False Discovery Rate: A Practical and Powerful Approach to Multiple Testing. Journal of the Royal Statistical Society: Series B (Methodological) 57:289–300. https://doi.org/10.1111/j.2517-6161.1995.tb02031.x

6. Lê Cao K-A, Boitard S, Besse P (2011) Sparse PLS discriminant analysis: biologically relevant feature selection and graphical displays for multiclass problems. BMC Bioinformatics 12:253. https://doi.org/10.1186/1471-2105-12-253
